# Effect of Czech Hop Varieties on Aroma of Dry-Hopped Lager Beer

**DOI:** 10.3390/foods11162520

**Published:** 2022-08-20

**Authors:** Kejda Tusha, Jakub Nešpor, Lukáš Jelínek, Hana Vodičková, Tomáš Kinčl, Pavel Dostálek

**Affiliations:** 1 Department of Biotechnology, Faculty of Food and Biochemical Technology, University of Chemistry and Technology, Prague, Technická 5, 166 28 Prague, Czech Republic; 2 Department of Chemistry and Chemical Education, Faculty of Education, Charles University, Magdalény Rettigové 4, 116 39 Prague, Czech Republic

**Keywords:** Czech hop varieties, beer, dry-hopping, SPME-GC-MS

## Abstract

The hoppy aroma in beer is characterized by an overall pleasant profile. The impacts of five Czech hop cultivars, Rubin, Saaz, Vital, Harmonie, and Kazbek, were tested on the hop aroma of the resulting beers, compared with a control beer sample, which was hopped with a commercial hop extract during wort boiling. GC-MS analysis was used for the identification and quantification of aroma-active compounds in the hops and beer. Dry hopping was successful in terms of improving the hoppy aroma in beer. Odorants such as β-myrcene, linalool, geraniol, β-citronellol, humulene epoxide I, and 2-methylbutyl-2-methylpropanoate were found at higher concentrations than the control in all dry-hopped beers. To quantify the success of dry hopping, the transfer rates of hop odorants were calculated. Those of linalool, geraniol and humulene epoxide I were adequate, whereas the transfer rates of polar compounds, e.g., β-myrcene, were relatively low, mostly due to their polarity. Changes in hop oil constituents were clearly notable, with the generation of β-citronellol, the release of other terpene alcohols from their glycosides or oxidation of α-humulene. Yeast metabolism also played an important role in these changes.

## 1. Introduction

Hop research is crucial for brewing science and technology, particularly regarding its importance for beer stability, preservation and, especially, organoleptic properties [1,2]. Originally, hops were used because some hop-derived compounds could inhibit the growth of microorganisms that cause beer spoilage [3]. Today, hops are chiefly added to beer to impart bitterness and a pleasant aroma [4]. It is essential for brewers, whether in craft or industrial breweries, to have a good methodological knowledge of hop composition and addition, in order to obtain a pleasant final product with particular properties, one of which, for instance, would be a beer with an enhanced hoppy aroma [1]. Several adjustments have been made to the brewing process, notably in the time, amount and method of hop addition, aiming to increase the hoppy aroma in beer and minimize the loss of hop active odorants during beer production [5].

The overall aroma in beer depends on volatile and non-volatile components found in or derived from the raw brewing materials [6]. The contribution of hops in the final aroma of beer has intrigued brewing chemists for years, as the chemistry, transfer, stability, and conversion of hop compounds imparting aroma are still not clear [1,4,7]. Several compounds responsible for the hoppy aroma in beer have been identified [7,8]. In fact, no single compound is responsible for the hop aroma; many of these compounds act in synergy and influence the final perception of aroma [9,10,11]. To use hops for brewing beers with an enhanced aroma, lupulin glands must contain a high ratio of α-humulene to β-caryophyllene (two sesquiterpenes found in hops responsible for aroma), which should be greater than 2.5 [12]. Generally, hops have been classified into two broad categories based on their main purpose: bittering and aroma.

Recent classifications put a specific focus on chemical composition, and divided bittering hops further into high alpha, and super high alpha varieties, based on the content of bitterness-imparting alpha acids [1,4]. Regarding hop oil composition (the hop fraction responsible for hop aroma), two subcategories should also be considered: fine aroma hops and aroma hops [1]. Some commonly used aroma varieties are Hallertauer Magnum, Hallertauer Taurus and Herkules from Germany; Galena and Tomahawk from the USA. Hallertauer Mittelfrüh, Hallertauer Perle, Hallertauer Tradition, Spalter Select and Tettnang Tettnanger from Germany; Vital, Saaz and Kazbek from the Czech Republic, and Cascade from the USA [1]. In Table 1 [13,14], are the listed characteristics of some of the aroma hop varieties available worldwide, and their most important chemical compositions: α-acids, responsible for bitterness, and essential oils, which are composed of odor-active compounds. Dry hopping differs thermodynamically from kettle and whirlpool hopping, as it is performed over a temperature range of 1–6 °C [15]. Dry hop cones or their derivatives impart bitterness and the characteristic aroma to the beer [16], and nowadays, dry hopping is the method primarily adopted for its influence on improving the aroma of beer [2,17]. In this paper, we tested the impacts of five Czech hop cultivars (Rubin, Saaz, Vital, Harmonie, and Kazbek) on the content of hop aromatic compounds in the dry-hopped beers. 

## 2. Materials and Methods

### 2.1. Chemicals

Diethyl ether (≥99.7%) and anhydrous sodium sulfate (≥99%) were purchased from Penta (Chrudim, Czech Republic). n-Hexane (≥99%, PESTINORM) and ethanol absolute (≥99.7 %, HiPerSolv CHROMANORM) were obtained from VWR (Darmstadt, Germany) and borneol from Merck (Darmstadt, Germany). Demineralized water was prepared using a Milli-Q Millipore water purification system Millipore (Bedford, MA, USA).

### 2.2. Hop Samples

Hop pellets, type 90 (produced from 2018 crop, growing region Saaz), from five Czech hop cultivars (Saaz, Rubín, Vital, Harmonie, Kazbek) were purchased from the Hop Research Institute, Saaz [14]. The hop pellets were sealed under vacuum and stored in a freezer before use.

### 2.3. Procedure for Dry-Hopped and Control Lager Beer Preparation

In an automated brewing system (100 L wort) at the University brewery, 17 kg of pale pilsner-type malt grist was mixed with 70 L of water and a decoction double mash system was used. Thirty-five liters of hot water was also used later for sparging. During wort boiling, only hop CO_2_ extract was added. After boiling, the wort was separated and cooled by wort cooler and transferred to the fermentation tank. Next, 800 mL of yeast slurry (15% dry weight, *Saccharomyces pastorianus*), purchased from brewery Budějovický Budvar n.p., was added to the fermentation tank. After primary fermentation at 8 °C, 60 L of beer was separated into six stainless steel kegs for maturation at 1 °C. Ten liters of beer containing only the hop extract was used as a control, whereas the other remaining five beer kegs were dry-hopped in addition to the hop extract added during wort boiling.

#### Procedure for Dry Hopping

For each hop cultivar, 10 L of beer was available. Based on sensory evaluation, which was previously carried out in our department, it was estimated that for 100 L of beer, 3 g of hop essential oils must be present. The calculated amount of hop pellets based on their oil content were placed in small sacks made of interwoven fabric (nylon + cotton), which were previously boiled in water. A static dry hopping method was applied. The sacks were placed in the beer kegs and after 30 days, the beer was transferred to plastic beer bottles, from which the beer was taken for further analysis.

### 2.4. GC-MS Analysis 

GC-MS analyses were performed on an Agilent 6890N GC gas chromatograph (Agilent Technologies, Santa Clara, CA, USA) coupled with a single quadrupole Agilent 5975B.

Inert MSD mass detector (Agilent Technologies, Santa Clara, CA, USA).

#### 2.4.1. Determination of Hop Essential Oils in Hop Pellets

##### Procedure for Isolation of the Oil Fraction Using Steam Distillation

The following procedure was applied for isolation of the essential oils in the five Czech hop cultivars. A measure of 100 g of hop pellets was weighed and then mixed with 2000 mL of distilled water in a 4000 mL round bottom boiling flask, which was heated in a heating mantle. The mixture was boiled and the distillation carried on for three hours. The distillate was collected every 30 min in a 100 mL narrow mouth Erlenmeyer flask and stored in the refrigerator. After 3 h of boiling, the heating mantle was turned off and the remaining distillate was collected. The condenser was then rinsed with a small amount of diethyl ether to collect small amounts of distillate entrapped on the walls of the condenser. The raw distillate was then purified twice by extraction with 25 mL of diethyl ether in a 250 mL separating funnel. The organic layer was collected and transferred to a 100 mL narrow mouth Erlenmeyer flask and dried overnight with anhydrous sodium sulfate in the refrigerator. After this, the dried solution was filtered to remove the anhydrous sodium sulfate, followed by evaporation under vacuum in a rotary evaporator at 35 °C to remove the organic solvent, until a constant weight of essential oils was reached.

In a 10 mL volumetric flask, 100 μL of essential oils was mixed with 100 μL of borneol in hexane (c = 71.96 g/L) and added up to the mark with hexane. The volumetric flask was vortexed and an aliquot of this solution was then transferred to a vial and further analyzed by GC-MS.

##### Conditions for GC-MS

– Carrier gas: helium (1 mL/min);
– Capillary column: HP-5MS (30 m × 0.25 mm, coating width 0.25 μm);
– Temperature program: 60 °C (5 min) → 2 °C/min →150 °C → 5 °C/min → 220 °C (5 min) → 20 °C/min → 300 °C (5 min);
– Injection: 1 μL of sample, split 1:30, T = 260 °C;
– MS conditions: scan mode, *m*/*z* = 35–350;
– Length of analysis: 88 min.

##### Data Evaluation

Borneol (c = 71.96 g/L) was used as an internal standard for quantitative analysis of hop essential oils. Based on the retention times and *m*/*z* ratio, the identification of individual components was carried out, following the manual integration of the peaks in the chromatogram in order to quantitatively calculate peak areas and the relative amount of these components.

#### 2.4.2. Determination of Hop Essential OILS in Beer

##### Procedure for Isolation of the Oil Fraction Using Steam Distillation

Beer from bottles was gravity filtered in a funnel with filter paper, a piece of cotton and a spoonful of kieselguhr. A measured volume of beer was then added to the boiling flask together with a pipetted amount of silicone antifoam, where it was steam-distilled for approximately 3 h. The distillate was collected in three different receivers. To the first receiver, 5 mL of denatured ethanol was added, to the second, 50 mL, and to the third, 10 mL. The second receiver was placed in a beaker filled with ice and the third one in a thermos filled with ice and sodium chloride. Approximately 310 mL of distillate from all three receivers was transferred into 600 mL glass bottles, into which 100 μL of borneol (c = 0.75 g/L) was added. The distillate from the beer matrix was then extracted using SPE. An SI-1 single-use column was used for pre-cleaning the distillate and a C_18_ single-use column was used to trap the essential oils in the sorbent. The columns were preconditioned, respectively with 5ml of n-hexane Pestinorm plus 5 mL of distilled water for SI-1 and 5 mL of ethanol absolute plus 5 mL of distilled water for C_18_. The columns were connected together and then with the distillate bottle through a rubber tube. In total, SPE lasted 3–4 h. 

The next task was to isolate the entrapped essential oil fraction in the sorbent of the C_18_ column. This was achieved by eluting the column with 6 mL of n-hexane Pestinorm under vacuum. The eluate was then transferred to a new 100 mL narrow mouth Erlenmeyer flask and dried overnight with anhydrous sodium sulfate. The next day, the eluate was filtered from the anhydrous salt in a PTFE disc filter with n-hexane Pestinorm, followed by evaporation under vacuum in a rotary evaporator. The remaining fraction was then transferred to a vial and topped up to 1.5 mL with hexane, ready for GC-MS analysis.

##### Conditions for GC-MS

– Carrier gas: helium (1 mL/min);
– Capillary column: HP-5MS (30 m × 0.25 mm, coating width 0.25 μm);
– Temperature program: 40 °C (3 min) → 5 °C/min → 220 °C → 20 °C/min → 300 °C (5 min);
– Injection: 1 μL of sample, split 1:30, T = 280 °C;
– Length of analysis: 49 min.

##### Data Evaluation

Borneol (c = 0.75 g/L) was used as an internal standard for GC-MS analysis. Individual components were identified according to the retention times and *m*/*z* ratio, using the internal standard calibration method. Peaks of the components of interest in the chromatogram were manually integrated, followed by a calculation of their relative levels.

## 3. Results

### 3.1. Analysis of Hop Pellet Essential Oils

The hop essential oil content of the five Czech cultivars Rubin, Saaz, Vital, Harmonie and Kazbek were determined 0.90, 0.61, 0.75, 0.84 and 1.10, respectively (wt. %). The list of compounds determined by GC-MS analysis is shown in Table A1, and attached in the Appendix A. The amount of each compound was calculated according to the internal standard method:

(1) $$c_{i}=\frac{A_{i}}{A_{\mathrm{standard}}}\times c_{\mathrm{standard}}\times 100\times \frac{m_{\mathrm{oil}}}{\rho_{\mathrm{oil}}}\;\left[\mu g/100\;g\;\mathrm{hops}\right]$$

Each compound in Table A1 is given as its relative mass percentage (rel.%) according to the total essential oil content:

(2) $$\mathrm{rel}.\%=\frac{c_{i}}{c_{\mathrm{total}}}\times 100$$

**Figure 1:**
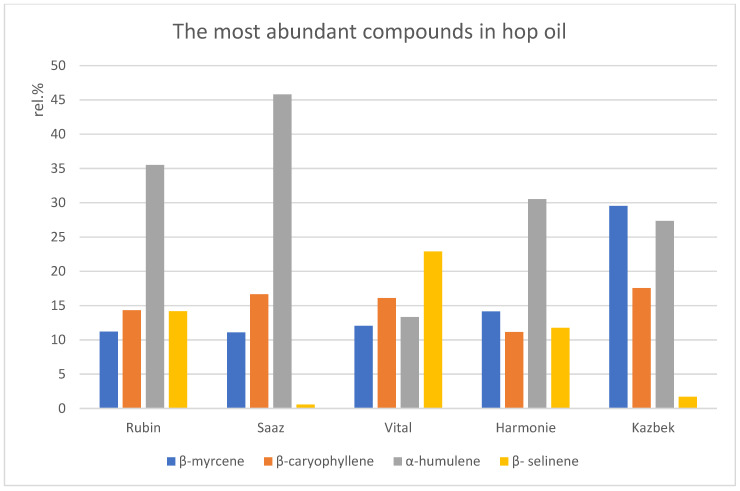
The most abundant compounds in the hop oil of five Czech hop cultivars.

The aroma-active compounds β-myrcene, β-caryophyllene, and α-humulene were dominant in all cultivars, with the exception of β-selinene, which was present at a more considerable content in Rubin, Vital and Harmonie cultivars (Figure 1).

#### Hop Addition for Dry Hopping

The amount of hop pellets added to the beer kegs was calculated based on the content of hop essential oils. Taking into account the sensory evaluation of previous work in our department, it was estimated that for 100 L of beer, 3 g of hop essential oil must be present. According to this relationship, the amount of hop pellets added was calculated with respect to their hop oil content and the fact that for each cultivar, 10 L of beer were available. We added to each 10 L of beer 33, 50, 38, 38 and 27 g of hop pellets, type 90, of different Czech cultivars Rubin, Saaz, Vital, Harmonie and Kazbek, respectively.

### 3.2. Analysis of Hop Oil in Beer

GC-MS analysis revealed the presence of some hop oil-derived compounds in beer, as presented in Table 2. The concentration of each component is given according to its total concentration in the analyzed beer, calculated using the internal standard method. Table 2 lists the original hop oil compounds, together with other hop-oil-derived components, such as β-citronellol. To assess the success of dry hopping, the transfer rates (TR) valid for 1 L of dry-hopped beer, as shown in Table 3, were calculated according to this equation, from which the values of the control beer were subtracted:

(3) $$\mathrm{TR}\;\left(\%\right)=\frac{c_{\mathrm{oil}\;\mathrm{in}\;\mathrm{dry}-\mathrm{hopped}\;\mathrm{beer}\;\left(\mu g/l\right)}-{\;c}_{\mathrm{oil}\;\mathrm{in}\;\mathrm{control}\;\mathrm{beer}\;\left(\mu g/l\right)}}{c_{\mathrm{oil}\;\mathrm{in}\;\mathrm{hops}\;\left(\mu g/g\right)}\times m_{\mathrm{hops}\;\mathrm{used}\;\left(g\right)}}\times 100$$

## 4. Discussion

As shown in Table 2, dry-hopped beers had a higher content of hop oil compared to the control beer, even without the fraction transferred from the hop extract. Hop-derived compounds such as 2-methylbutyl-2 methylpropanoate, limonene, geraniol and methyl geranate were not found in the control beer. Even though limonene was present at trace levels in all dry-hopped beers, due to its coelution with other compounds in the chromatographic peak, its amount could not be quantified. The same pattern followed for geraniol, methyl geranate, caryophyllene oxide and α-cadinol for some varieties. In comparison with the control beer, dry-hopped beers had a much higher content of β-myrcene, linalool, β-citronellol, 2-undecanone, humulene epoxide I and 2-dodecanol.

As has been shown in previous reports, the linalool content in dry-hopped beers increased due to its high solubility in beer, thus affecting its transfer rate (Table 3) from hop pellets [18,19]. Its increase does not depend on the cultivar, as in all dry-hopped beers the linalool content was high, with transfer rates between 44 and 81%. This non-varietal dependence was also shown in a previous report [20]. The linalool transfer rate even exceeded 100% in some cases, but its increase in beer could be attributed to the release of linalool from linalool glycosides or the isomerization of nerol and geraniol to linalool, two types of biotransformations induced by yeast metabolism [20,21,22]. Another monoterpene alcohol that could be affected by yeast metabolism is geraniol. Geraniol was present in all cultivars, with the highest relative mass percentage of 0.53% in Kazbek. In the dry-hopped beer with Kazbek, the geraniol transfer rate exceeded 100%. In comparison, its transfer rate in Vital beer was only 22.4%. Because of coelution with other non-hop derived compounds during chromatographic separation, the precise content of geraniol could not be calculated in the dry-hopped beers with Rubin, Saaz, and Harmonie cultivars. A possible reason for the transfer rate in Kazbek beer could be the existence of some glycosidically bound terpene alcohols such as nerol glycoside or geraniol glycoside, which, upon yeast enzyme activity, release geraniol into the beer [23,24]. The occurrence of β-citronellol in all beers is an indicator that once again, yeast metabolism strongly affected the hop oil content. The generation of β-citronellol was due to geraniol reduction, but the possibility of the existence of a glycosidically bound precursor is also valid [22,25].

The coexistence of linalool, geraniol and β-citronellol, has an additive effect on the hoppy aroma in beer [25]. Linalool can enhance the odors of geraniol and β-citronellol, suggesting that not only linalool but also geraniol and β-citronellol are odor-active compounds [25]. As these three monoterpene alcohols are present at considerable levels in these dry-hopped beers, they play a positive role on the hoppy aroma.

Regarding terpenes, their transfer rates in dry-hopped beers were lower compared to terpenoids. The content of β-myrcene was higher in the dry-hopped beers compared to the control beer. Those of linalool, geraniol and humulene epoxide I were adequate, whereas the transfer rates of non-polar compounds, e.g., β-myrcene, were relatively low, mostly due to their polarity. [19]. Furthermore, β-myrcene can also undergo auto-oxidation, yielding α- and β-pinene, or terpenoids such as linalool, nerol or geraniol [26,27,28]. In parallel, the high content of these terpene alcohols in dry-hopped beers could also be attributed to derivatives of myrcene. Another reason for the low transfer rate could also be adsorption from the plastic corks of the beer bottles [29]. α-Humulene was not detected in any of the beers and one possible reason may be its oxidation to humulene epoxide I [30]. Physical factors should be taken into account as well because there is a possibility that α-humulene and its oxidation products could have been adsorbed onto the yeast biomass [31]. Although β-caryophyllene was present in a considerable amount in all hop cultivars, it was not found in any of the dry-hopped beers. Its oxidized form, caryophyllene oxide, was quantified in Vital and Kazbek beers [32].

## 5. Conclusions

The potential of dry hopping was confirmed when producing beers with an enhanced hoppy aroma. Odorants responsible for this aroma profile, mostly the polar ones, were transferred from hop pellets to beer at levels that greatly surpassed their flavor thresholds known from literature [7]. The effect of yeast on the levels of these polar compounds was clearly noted. Some advantageous changes included the release of monoterpene alcohols from their glycosides and their isomerization or reduction to more odorous compounds, such as linalool and β-citronellol. Compared to the control beer, dry-hopped beers also contained a higher concentration of non-polar compounds, although their transfer rates were not high due to their low polarity.

In agreement with previous works, hop oil constituents underwent many changes that could not be avoided by dry hopping. Autooxidation, yeast-induced biotransformations and biomass adsorption caused losses of the non-polar terpene fraction. However their oxidation products, e.g., humulene epoxide I and caryophyllene oxide, are favorable odor-active components. This study adds relevant information to the brewing industry for the use of these Czech varieties of hops for lager dry-hopping.

## Figures and Tables

**Table 1:** The main characteristics of selected aroma hop varieties [13,14].

Cultivar	Country	Aroma	α-Acid Content (wt. %) *	Total Oil (mL/100g)	Myrcene (rel.%)	α- Humulene (rel. %)	β- Caryophyllene (rel.%)	H:C (Ratio)
Amarillo	USA	Floral, citrus	7–11	1–2.3	40–50	19–24	7–10	1.9–3.4
Citra	USA	Citrus, tropical fruit	11–15	1.5–3	60–70	7–12	5–8	0.9–2.4
Cascade	USA	Floral, citrus, spicy	5.5–9	0.8–2.5	45–60	14–20	5–9	1.5–4
Nelson Sauvin	New Zealand	Fruity	12–13	1.1	22–23	36.4	10.70	3.4
Hallertauer Mittelfrüh	Germany	Floral, citrus, spicy	3–5.5	0.7.1.3	14–16	55–55.2	14.5–14.7	3.7–3.8
Tettnang Tettnanger	Germany	Spicy, herbal	3–6	0.5–0.9	20–35	20–30	6–11	1.8–5
Spalter Select	Germany	Grassy, spicy	3–6.5	0.6–0.9	20–40	10–22	4–10	1–5.5
Vital	Czech Republic	Spicy	12–16	1.8–2.9	40–60	2–5	5–8	0.25–1
Saaz	Czech Republic	Floral, citrus, spicy, herbal	3–5	0.5–0.9	25–40	15–25	5–8	1.9–5
Kazbek	Czech Republic	Spicy, lemon	5–8	1.06–2.1	40–55	20–35	10–15	1.3–3.5
Wye Challenger	UK	Fruity, herbal, cedar	6.5–8.5	1–1.7	30–42	5	9.5	2.6

* Weight percentage based on total dried hop cones.

**Table 2:** Content of hop essential oil compounds in beer.

Type of Beer	Control	Rubin	Saaz	Vital	Harmonie	Kazbek
**Determined compounds**	Concentration in beer (μg/L)					
**β-myrcene**	7	101	23	61	78	75
**2-methylbutyl-2-methylpropanoate**	N.D.	59	8	47	29	35
**limonene**	N.D.	*	*	*	*	*
**2-nonanone**	N.D.	N.D.	N.D	45	N.D.	N.D.
**linalool**	25	146	83	321	323	186
**nonanal**	18	N.D.	20	N.D.	N.D.	N.D.
**β-citronellol**	17	37	28	40	37	64
**geraniol**	N.D.	*	*	22	*	179
**2-undecanone**	11	57	56	84	60	35
**2-dodekanol**	6	35	23	48	30	133
**methyl geranate**	N.D.	153	*	118	247	*
**α-humulene**	N.D.	N.D.	N.D.	N.D.	N.D.	N.D.
**(E)-nerolidol**	20	59	25	34	38	75
**caryophyllene oxide**	*	*	*	100	*	137
**humulene epoxide I**	262	383	499	299	493	405
**α-cadinol**	*	*	94	*	*	*
**farnesol**	30	73	34	34	63	51
**SUM**	**397**	**1104**	**893**	**1252**	**1403**	**1258**
**Control beer-subtracted SUM**	**-**	**707**	**496**	**855**	**1006**	**861**

N.D.—not detected. *—compound present in a non-quantifiable amount.

**Table 3:** Transfer rates of hop essential oils to beer.

Cultivar	Rubin	Saaz	Vital	Harmonie	Kazbek
**Determined compounds**	**TR (%)**				
**β-myrcene**	3.4	0.6	2.4	1.6	0.8
**2-methylbutyl-2-methylpropanoate**	31.3	24.4	15.8	57.1	21.9
**2-nonanone**	N.D.	N.D.	27.4	N.D.	N.D.
**linalool**	80.2	52.0	44.3	69.8	55.4
**geraniol**	N.D.	N.D.	22.4	N.D.	120.4
**2-undecanone**	41.3	11.1	5.6	12.3	17.4
**methyl geranate**	54.2	N.D.	28.0	59.5	0
**(E)-nerolidol**	N.D.	N.D.	N.D.	N.D.	54.0
**caryophyllene oxide**	N.D.	N.D.	51.9	N.D.	55.53
**humulene epoxide I**	30.2	25.2	29.9	59.7	39.3
**α-cadinol**	N.D.	96.9	N.D.	N.D.	N.D.
**farnesol**	37.7	3.8	8.0	37.4	6.6

N.D.—not detected or compound at a non-quantifiable amount.

## Data Availability

Data are contained within the article.

## Appendix A

**Table A1:** Hop oil composition in five Czech hop cultivars.

Cultivar	Rubín	Saaz	Vital	Harmonie	Kazbek
Determined Compounds			Relative Mass Percentage (rel. %)		
**β-pinene**	0.24	0.21	0.95	0.27	0.73
**β-myrcene**	11.20	11.09	12.05	14.15	29.55
**2-methylbutyl-2-methylpropanoate**	0.77	0.13	1.62	0.16	0.58
**limonene**	0.13	0.11	0.48	0.24	0.36
**ocimene**	0.08	0.06	0.04	0.04	0.43
**2-nonanone**	0.08	0.20	0.88	0.21	N.D.
**linalool**	0.62	0.46	3.63	1.40	1.05
**nonanal**	N.D.	0.05	N.D.	N.D.	N.D.
**3-methylbutyl-3 methylbutanoate**	0.19	N.D.	0.54	0.03	0.06
**camphor**	0.30	0.30	0.37	0.24	0.29
**2-decanone**	N.D.	0.28	1.39	0.22	0.06
**methyl nonanoate**	N.D.	N.D.	0.24	0.06	0.01
**heptyl isobutyrate**	N.D.	N.D.	0.04	0.01	0.02
**geraniol**	0.18	0.12	0.53	0.08	0.54
**(E)-citral**	N.D.	0.01	0.04	0.02	N.D.
**2-undecanone**	0.45	1.65	7.06	1.30	0.48
**methyl decanoate**	0.38	0.29	1.51	0.28	0.04
**methyl geranate**	1.15	0.82	2.29	1.36	0.60
**α-cubenene**	N.D.	0.04	N.D.	N.D.	0.08
**ylangene**	0.15	0.17	0.20	0.16	0.20
**copaene**	0.65	0.78	0.42	0.60	0.63
**geranyl acetate**	N.D.	N.D.	N.D.	N.D.	0.10
**2-dodecanone**	0.09	0.23	0.95	0.12	0.11
**β-caryophyllene**	14.33	16.64	16.09	11.15	17.56
**β-cubenene**	0.44	0.37	0.31	0.29	0.49
**α-bergamotene**	N.D.	0.57	0.33	0.03	N.D.
**α-humulene**	35.53	45.79	13.33	30.52	27.34
**nerylacetate**	3.87	2.17	4.07	3.11	1.99
**γ-muurolene**	3.93	2.01	0.07	0.11	0.22
**β-selinene**	14.16	0.57	22.89	11.75	1.68
**α-selinene**	N.D.	N.D.	N.D.	13.92	2.51
**α-muurolene**	0.65	1.11	0.71	0.62	0.56
**geranyl butyrate**	N.D.	N.D.	N.D.	N.D.	1.36
**ν-cadinene**	1.93	2.51	1.02	1.28	1.78
**δ-cadinene**	3.03	3.79	2.21	2.55	3.13
**cadina-1,4-dien**	0.32	0.32	0.11	0.24	0.28
**α-cadinene**	0.36	0.44	0.14	0.31	0.34
**α-calacorene**	0.16	0.31	0.35	0.19	0.19
**(E)-nerolidol**	N.D.	N.D.	N.D.	N.D.	0.50
**caryophyllene oxide**	0.42	0.96	1.05	0.29	0.89
**humulene epoxide I**	1.63	3.87	0.67	1.27	1.31
**caryophyllene alcohol**	0.08	0.24	0.15	N.D.	0.16
**epi-bicyclosesquiphellandrene**	0.59	0.33	0.33	0.33	0.31
**copaene**	0.08	0.05	N.D.	0.04	0.07
**α-cadinol**	1.28	0.40	N.D.	N.D.	0.13
**pentadecanone**	0.05	0.21	0.62	0.12	0.03
**farnesol**	0.47	0.40	0.27	0.29	1.17
